# Trusting autonomous vehicles as moral agents improves related policy support

**DOI:** 10.3389/fpsyg.2022.976023

**Published:** 2022-10-20

**Authors:** Kristin F. Hurst, Nicole D. Sintov

**Affiliations:** ^1^School of Earth Systems and Sustainability, Southern Illinois University, Carbondale, IL, United States; ^2^School of Environment and Natural Resources, The Ohio State University, Columbus, OH, United States

**Keywords:** technology adoption, trust, autonomous vehicles (AVs), AV policy support, technology acceptance

## Abstract

Compared to human-operated vehicles, autonomous vehicles (AVs) offer numerous potential benefits. However, public acceptance of AVs remains low. Using 4 studies, including 1 preregistered experiment (total *N* = 3,937), the present research examines the role of trust in AV adoption decisions. Using the Trust-Confidence-Cooperation model as a conceptual framework, we evaluate whether perceived integrity of technology—a previously underexplored dimension of trust that refers to perceptions of the moral agency of a given technology—influences AV policy support and adoption intent. We find that perceived technology integrity predicts adoption intent for AVs and that messages that increase perceived integrity of AV technology result in greater AV adoption intent and policy support. This knowledge can be used to guide communication efforts aimed at increasing public trust in AVs, and ultimately enhance integration of AVs into transport systems.

## Introduction

Fully autonomous Vehicles (AVs) – those that use advanced technologies to sense the surrounding environment and navigate without human input – represent one of the most transformational advances in transportation technology. Compared to human-operated vehicles, AVs offer numerous potential benefits, including reduced fatal crash rates (Fagnant and Kockelman, 2015; National Highway Traffic Safety Administration, 2016) improved fuel efficiency, and reduced greenhouse gas emissions (Mersky and Samaras, 2016; Sun et al., 2016). Other benefits of AVs include the potential to reduce traffic congestion, regain time by doing activities other than driving, and providing mobility services to those with limited mobility, such as elderly and disabled individuals (West et al., 2003; Howard and Dai, 2014; Fagnant and Kockelman, 2015; Anderson et al., 2016; Bansal et al., 2016).

Despite these advantages the general public is still resistant to the widespread adoption and use of AVs (Haboucha et al., 2017), with the majority of potential AV riders expressing some level of worry about safety, security, and automation performance (Schoettle and Sivak, 2014; Pew Research Center, 2017). Similarly, a recent Gallup (2018) poll found that 47% of Americans believe the safest option is to have only or mostly human-operated vehicles on the road. Thus, willingness to use AVs remains limited, and further research is needed to understand how to overcome this public skepticism.

A growing body of research has begun identifying factors most likely to influence public acceptance of AVs. Key variables include cost, mobility behavior, demographics such as gender, income and education, prior knowledge, experience and familiarity, and perceived risks and benefits (Nordhoff et al., 2019; Alawadhi et al., 2020; Golbabaei et al., 2020; Peng, 2020; Acheampong et al., 2021; Rahman et al., 2021). Additionally, accumulating evidence suggests that trust in AVs is a critical determinant of whether the public will accept this emerging technology (e.g., Shariff et al., 2017; Dixon et al., 2018; Liu et al., 2018; Yuen et al., 2020; Waung et al., 2021). For example, Shariff et al. (2017) argue that understanding how to promote the formation of “trustable mental models” of AVs among the public will be critical for overcoming psychological barriers to AV adoption (p. 694). Recent empirical research on trust and AV acceptance by Yuen et al. (2020) found—among other insights—that the relationship between a person’s perceived value of AVs and acceptance is partially mediated by trust. Additionally, a study by Waung et al. (2021) explored trust in several trustees in the AV domain: AV manufacturers, AV regulators, and in the technical capabilities of AVs. They found that trust in AVs’ technical capabilities mediated relationship between perceived risks of AV failure and behavioral intentions to ride in AVs.

In the context of trust in AVs, we argue that it is important to attend to two factors. First, there may be multiple potential trustees involved, including (1) the technology or vehicle itself as well as (2) various agents—that is, individuals or organizations responsible for manufacturing, regulating, or otherwise overseeing the technology (Waung et al., 2021). Second, per the Trust-Confidence-Cooperation model (TCC), multiple dimensions of trust exist, including (1) confidence, or competence, and (2) integrity (Earle and Siegrist, 2006, 2008; Siegrist, 2019); these dimensions should be assessed and considered separately (see section “Dimensions of trust per the Trust-Confidence-Cooperation model: Competence and integrity” below for details). Thus, a person may ascribe both competence and integrity to both types of trustees- the technology itself and agents overseeing it. However, an abundance of research asserts that integrity can only be conferred upon agents and not upon technology itself, and/or does not measure integrity of the technology (e.g., Earle et al., 2010; Siegrist, 2019). Hence, the integrity of the technology has been implicitly – and sometimes explicitly – overlooked. The present research challenges this assumption regarding perceived integrity of the technology.

Informed by the TCC model, the goal of the current research is to advance understanding of the relationship between trust and AV acceptance by examining an aspect of trust that has largely been overlooked: perceived integrity of the AV technology. In the following section, we review prior empirical and theoretical literature on this topic and explain why our focus on technology integrity fills an important research gap.

### Trust and automation

Research across and within academic disciplines has long used a variety of definitions for trust (Rotter, 1971; Mcknight et al., 1996; Grabner-Kräuter and Kaluscha, 2003; Colquitt et al., 2007; Thielmann and Hilbig, 2015). Broadly, however, trust refers to the willingness of an individual (the trustor) to rely on another actor or object (the trustee) in situations characterized by uncertainty and vulnerability (Lee and See, 2004). Extensive research has identified trust as a key factor guiding behavior and decision-making in human-automation interactions. As we highlighted above, this includes research specific to the AV context.

Factors that determine whether a person will trust automation include those related to (1) the trustor (e.g., individual traits, dispositions, psychological states), (2) the situation or environment (e.g., social context, level of risk, organizational setting) or, (3) the trustee (e.g., past performance or appearance) (Hoff and Bashir, 2015; Schaefer et al., 2016). Regarding the trustee, prior research has identified a set of key characteristics related to the automation itself that can influence perceptions of trust. These include the features of the automation such as the mode or style of communication, the level of automation and the appearance of the technology (e.g., whether it appears human-like) (Schaefer et al., 2016). Broadly, this research has found that people are more likely to trust automation that has human-like features and appearance (e.g., Pak et al., 2012; De Visser et al., 2016), communicates politely with users (Parasuraman and Miller, 2004; Spain and Madhavan, 2009) and is easy to use (Atoyan et al., 2006). In addition, the performance and reliability of automation is critical to trust formation. Quite simply, automation that can be consistently relied upon to perform as expected will be trusted by users (Hoff and Bashir, 2015; Schaefer et al., 2016).

### Dimensions of trust per the Trust-Confidence-Cooperation model: Competence and integrity

The latter set of features aligns closely with a key dimension of trust outlined in the TCC model termed confidence, or competence-based trust (Rousseau et al., 1998; Siegrist et al., 2005, 2012; Earle and Siegrist, 2006, 2008; Visschers and Siegrist, 2008; Earle, 2010; Siegrist and Zingg, 2014; Siegrist, 2019; Liu et al., 2020). Competence-based trust is **“**based on past experiences or evidence suggesting that future events will occur as expected” (Siegrist, 2019, p. 483). In other words, competence-based trust relates to the willingness to rely on a person or technology due to performance and ability beliefs (Earle and Siegrist, 2006; Allum, 2007; Terwel et al., 2009; Siegrist, 2019). Because failure of AV technology carries a risk of fatality, trust in the technical capabilities of AVs is key; people need to trust that the AV will perform as expected. Overall, research supports positive relationships between competence-based trust (and related constructs) and technology use intention (e.g., Grabner-Kräuter and Kaluscha, 2003; Pavlou, 2003). For conciseness, throughout the remainder of the manuscript, we simply refer to this concept as “competence” rather than competence-based trust.

However, the TCC model stipulates a second and conceptually distinct dimension of trust termed relational trust, or integrity-based trust (Earle and Siegrist, 2006, 2008; Siegrist, 2019). Integrity-based trust is “based on the judgment of similarities in intentions and values” (Siegrist, 2019, p. 483). In other words, this dimension of trust refers to the willingness to rely on relevant others due to perceptions of shared values and good intentions on the part of the trustee. For conciseness, throughout the remainder of the manuscript, we simply refer to this concept as integrity, rather than integrity-based trust. In the case of AVs, and perhaps automation more broadly, it is not just competence that matters. People need to trust that AV vehicle manufacturers and government authorities that regulate vehicle production are operating in honest and transparent ways and have the best interest of the public in mind (Earle and Siegrist, 2006; Liu et al., 2020). Hence, distinct from the ability of a technology to perform as intended (i.e., competence), integrity indicates to what extent people are willing to rely on relevant others and institutions. Overall, prior work supports a positive relationship between integrity and acceptance of technology, including AVs (Siegrist, 1999; Liu et al., 2018) (see Appendix A for a conceptual diagram of the TCC model).

### Types of trustees: The technology vs. the social entity

The importance of trusting not just the technology itself, but also the social entities associated with and responsible for the technology (e.g., manufacturer, regulator, designer), is acknowledged to some degree in the automation literature. For example, in a 2015 review of the trust in automation literature, Hoff and Bashir state that trust in a technological system is, to a degree, representative of the trust one has in the system’s designers. However, they go on to argue that there is an important distinction between trust in a social entity (e.g., the designer, in Hoff and Bashir’s language), and the technology itself such that trust in a social entity is generally “based on the ability, integrity or benevolence of the trustee [whereas] human-automation trust depends on the performance, process, or purpose of an automated system” (p. 11). The TCC literature makes a similar claim. Namely, scholars have argued that a key distinction between competence and integrity is that whereas competence can be conferred upon a person, organization, or object (e.g., “I trust the AV manufacturer to have the technical expertise to make safe cars.” Or, “I trust the AV to perform as expected”) (Earle et al., 2010; Siegrist, 2019), integrity implies agency or intentions on the part of the trustee and, thus, can only be conferred upon a person or a “person-like” entity such as an institution (i.e., “I trust the AV manufacturer to have the best interest of the public in mind”). Critically, in other words, integrity is typically only conferred upon an agent (a person or organization) and not an object, such as a car, washing machine, or other form of technology. In a 2019 review of the trust literature, Siegrist explains this distinction:

“From a phenomenological perspective, trust in an institution or a person differs from that in an object, such as a washing machine or a car. Some authors adopt both phenomena to define trust (Shapiro, 1987), but this means ignoring significant differences in people’s experiences. Although a washing machine or a car may not function as expected, they certainly will not deceive people because these objects lack the intention to do so” (p. 5).

In the present research, we challenge the assumption embedded in both the automation literature and the TCC model that people do not imbue technology with integrity and instead propose that, at least in the case of AVs, public perceptions of the competence and integrity of AV technology impacts AV acceptance. Unlike other forms of technology—including non-autonomous vehicles, which people maintain a high degree of control over while operating, and airplanes, in which passengers cede control to another human operator (i.e., the pilot) —AVs act as autonomous agents in which passengers entrust their lives and safety. Prior work on mind perception and morality has found that the ascription of moral responsibility is a matter of perception that depends to a large extent on agency (Gray et al., 2007, 2012; Bigman et al., 2019). That is, in order to hold someone or something morally responsible a person must perceive that they have the ability to plan, remember, make decisions and communicate. Because autonomous technologies, such as AVs, may be seen to have these characteristics, people may be more likely to reason about them as moral agents than they are human-operated technology (Waytz et al., 2014; Bigman et al., 2019). As such, despite a lack of attention to the perceived integrity of the technology in prior literature, it seems plausible that people ascribe not only competence, but also integrity to AV technology—trusting the vehicles, for example, to “do the right thing,” keep people safe in unexpected circumstances, and “communicate openly” with their passengers about potential dangers. Thus, the overarching purpose of the research is to examine the relationship between technology integrity—a previously underexplored dimension of trust— and AV acceptance (i.e., adoption intent and policy support) relative to other theoretically and empirically established dimensions of trust.

### Study objectives and contributions

Before examining integrity of AV technology, the first objective of this research was to determine the extent to which trust in AV technology broadly (i.e., not broken down into the previously described dimensions of integrity and competence) influences AV decision making above and beyond the effects of other key predictors such as risk, affect, and perceived benefits (Visschers and Siegrist, 2008; Choi and Ji, 2015; Liu et al., 2018). We then build on work based on the TCC model by evaluating whether perceived integrity of technology uniquely influences AV policy support and adoption intent. To do this, we examine two dimensions of trust (competence and integrity) in two entities (AV technology and AV manufacturers). Thus, we examine: (1) competence of AV technology, (2) integrity of AV technology (3) competence of AV manufacturers, and, (4) integrity of AV manufacturers. Of these, integrity of the AV technology has been overlooked in prior research. This research advances understanding of barriers to public acceptance of AVs and can be used to enhance integration of AVs into transport systems.

### Overview of current research

We conducted four studies. Study 1 (*N* = 455) applies hierarchical modeling to survey data to evaluate whether trust in AV technology explains unique variance in AV policy support and adoption intent above and beyond a set of established variables known to influence these outcomes. Next, Studies 2a (*N* = 1,691) and 2b (*N* = 853) use surveys to evaluate the influences of integrity and competence ascribed to both the AV manufacturer and the technology itself on adoption intent in two domains (AVs and airplanes). Finally, in Study 3 (*N* = 938), which was a 2 (integrity: high vs. low) × 2 (competence: high vs. low) × 2 (trustee: AV manufacturer vs. AV technology) between-subjects experiment pre-registered at 10.17605/OSF.IO/A7RZT,^1^ we experimentally test whether manipulating perceptions of integrity and competence of AV technology as well as manufacturers increases AV policy support and adoption intentions.

All studies were deemed exempt by The Ohio State University Institutional Review Board (Protocols 2019E0273, 2020E0497, 2020E0359). All studies recruited participants from online research platform Prolific.co. Prolific participants have been found to be more demographically diverse, more naïve, and less dishonest than (MTurk) participants (Peer et al., 2017). Eligibility criteria included U.S. as current country of residence and age of at least 18 years.

## Study 1

### Overview

The purpose of Study 1 was to establish the extent to which trust in AV technology factors into AV decision making. Despite the bulk of the evidence supporting a positive direct relationship between trust and technology acceptance, the extent to which trust explains unique variance in technology adoption outcomes when parceling out variance accounted for by other predictors remains unclear. It is possible that when a adjusting for the effects of other key predictors such as risk, affect, and perceived benefits (Visschers and Siegrist, 2008; Choi and Ji, 2015; Liu et al., 2018), trust itself makes no unique contribution to AV acceptance. Thus, the purpose of Study 1 was to test the unique influence of trust in AV technology on adoption decisions, above and beyond a set of established predictors.

### Methods

#### Procedures

Respondents completed an online survey on Qualtrics in Spring 2019. The survey took approximately 20 min, and participants were compensated $2.70. Before responding to survey measures, participants read a definition of AVs adapted from definitions provided in Choi and Ji (2015) and Zhang et al. (2019) (Appendix B; the same definition was also presented in Studies 2a, 2b, and 3). Participants then completed a series of measures of AV adoption intention and policy support, followed by measures of trust, affect, perceived benefits, and knowledge related to AVs. Several covariates were also assessed, including AV experience, knowledge and demographics. Three attention check items (e.g., “Please select ‘never’ as your response to this item”) were included. For all scale variables that we created, scale scores were given if respondents answered at least two-thirds of the questions used to form the scale. Otherwise, the scale variable was coded as missing.

#### Measures

##### Trust in autonomous vehicle technology

Trust was measured with a single item, “I would trust an AV,” assessed on a 6-point scale ranging from 1 = Strongly Disagree, to 6 = Strongly Agree.

##### Autonomous vehicle adoption intention

Intention to use AVs was measured using four items adapted from Choi and Ji (2015) and White and Sintov (2017) in which participants rated their responses on a five-point Likert scale (1 = strongly disagree to 5 = strongly agree) (e.g., “I intend to use AVs in the future;” “I intend to buy an AV in the future”). A scale (Cronbach’s α = 0.95) was formed by taking the mean.

##### Autonomous vehicle policy support

Policy support for AVs was measured using five items adapted from Dixon et al. (2018) in which participants rated their responses to the question “To what extent do you support/oppose the following actions?” on a six-point bipolar scale (1 = strongly oppose to 6 = strongly support) (e.g., “The sale of AVs in the United States;” “The increased use of AVs in the United States”). A scale (Cronbach’s α = 0.96) was formed by taking the mean.

##### Perceived risk

To measure perceived risks of AVs, participants rated a set of eight items adapted from Liu et al. (2018) (e.g., “I am concerned about AVs sharing the roads with human-driven vehicles”) on a five-point Likert scale (1 = strongly disagree to 5 = strongly agree) (Cronbach’s α = 0.74).

##### Autonomous vehicle-related affect

Affect toward AVs was measured with a single item adapted from Dixon et al. (2018) in which participants rated how they felt when thinking about AVs on a seven-point bipolar scale (1 = very bad to 7 = very good).

##### Perceived benefits of autonomous vehicles

Perceived benefits of AVs were assessed with the five questions also used in (Liu et al., 2018): “AVs can reduce traffic congestion,” “AVs can reduce vehicle emissions and pollution,” “AVs can improve fuel economy,” “AVs can reduce transport costs,” and “AVs can increase the mobility of those who are currently unable to drive.” This question was rated on a 6-point Likert scale (1 = Strongly Disagree to 6 = Strongly Agree, no neutral option) (Cronbach’s α = 0.85).

##### Autonomous vehicle knowledge

AV knowledge was assessed using eight true/false items adapted from Sanbonmatsu et al. (2018) (e.g., Fully autonomous vehicles will rely heavily on GPS for navigation). The true/false answers were then scored as either correct or incorrect. The final knowledge variable is calculated as the percentage of answers that respondents answered correctly.

##### Autonomous vehicle experience

Autonomous vehicle (AV) experience was assessed by having participants check types of vehicle automation they have used (e.g., vehicle with cruise control, fully autonomous vehicle). Responses were coded “1” for AV experience if they have experience any of the more complex forms of automation, including: a fully autonomous vehicle, a vehicle with lane assist, a vehicle with automatic braking, or a vehicle with automatic parking. Responses were coded “0” for no experience with automation or only experience with more common and longer-established forms of automation, i.e., cruise control only.

##### Demographics

Participants also answered a range of demographic questions.

##### Age

We coded age as 2019 minus participants’ answers to what year they were born.

##### Education

Participants indicated their highest level of education (including education in progress), with response options as follows: did not complete high school, high school/GED, some college/associate’s degree, 4-year college degree, or graduate degree. We coded education as a dichotomous variable split at the median. Respondents were coded 1 if reported that they had 4-year college or greater, and otherwise coded 0.

##### Gender identity

Participants were asked to select a gender that most closely represented their gender identity (1 = male, 0 = female or other^2^).

##### Income

Participants indicated their annual household income, with response options as follows: less than $10,000, $10,000-$14,999, $15,000-$24,999, $25,000-$34,999, $35,000-$49,999, $50,000-$74,999, $75,000-$99,999, $100,000-$149,999, $150,000-$199,999, or $200,000 or more. We coded income as a dichotomous variable split at the median, such that respondents were coded 1 if their income was $50,000 to $74,999 or higher, and 0 otherwise.

##### Political orientation

Political orientation was measured using a seven-point scale that ranged from 1 = very liberal to 7 = very conservative.

#### Participants

From our initial sample of 500, we dropped 1 participant who did not complete the survey, and 19 who failed any one of the three attention checks. Among the remaining 480 respondents, we dropped 14 individuals each from the top and bottom 2.5% of survey duration, due to concerns that atypically fast or slow speeds may indicate lack of attention or distraction (Greszki et al., 2015). This left us with a final sample of *N* = 455 individuals. The median age of participants was 32 years old. Roughly 48% identified as women. Nearly half reported receiving a bachelor’s degree or higher (49%). The sample leaned liberal, with 61% of participants identifying as at least slightly liberal.

### Results

We ran hierarchical regression models to determine whether trust in AV technology explains unique variance in AV policy support, above and beyond other predictors. Independent variables included in the first three blocks are as follows: (1) demographics (age, gender, income, education level, political orientation); (2) familiarity (AV experience, AV knowledge); and (3) key variables (risk perceptions, perceived benefits, affect). In the fourth block we added trust (Table 1).

**TABLE 1:** Study 1 hierarchical regression models predicting autonomous vehicle (AV) policy support (*N* = 455).

	Model I	Model II	Model III	Model IV
Constant	4.86***	3.95***	0.39	0.34
**Block 1: Demographics**				
Gender (man)	0.54***	0.48***	0.05	0.04
Age	−0.02***	−0.02***	0.001	0.001
Income	0.32*	0.25^+^	0.01	0.006
Education	0.12	0.05	0.09	0.02
Political Orientation^1^	−0.17***	−0.17***	−0.07***	−0.07***
**Block 2: Familiarity**				
AV experience		0.23^+^	0.07	0.05
AV knowledge		1.16**	0.25	0.19
*R^2^ change*		0.02		
*F* (2,447)		5.55*		
**Block 3: Key variables**				
Risk perceptions			−0.18***	−0.09^+^
Perceived benefits			0.40***	0.27***
AV-related affect			0.57***	0.34***
*R^2^ change*			0.63	
*F* (3,444)			430.42***	
**Block 4: Trust**				
Trust				0.36***
*R^2^ change*				0.03
*F* (1, 443)				82.76***
**Omnibus**				
R^2^	0.13	0.15	0.78	0.82
F (df)	*F* (5,449) = 13.65***	*F* (7, 447) = 11.54***	*F* (10,444) = 160.47***	*F* (11,443) = 180.27***

***p < 0.001, **p < 0.01, *p < 0.05, ^+^*p* < 0.10. ^1^ Political orientation was measured on a 7-point scale with 1 = very liberal and 7 = very conservative.

We found that the variables in block 1 (i.e., demographics) explained 13% of the variance in AV Policy Support [*R*^2^ = 0.13, *F* (5,449) = 13.65, *p* < 0.001]. The second block, which added AV experience and knowledge explained an additional 2% of variance above the demographic variables [*R*^2^ *change* = *0.02, F change* (2,447) = 5.55, *p* < 0.05]. Next, we added perceived risks, perceived benefits and affect in block 3. Adding these key variables increased the variance explained by 63% [*R*^2^ *change* = 0.63, *F change* (3,444) = 430.42, *p* < 0.001]. Finally, above and beyond factors representing demographic characteristics, familiarity, perceived risks, perceived benefits, and affect, trust explained an additional 3% of the variance in AV policy support [R^2^ *change* = 0.03, *F change* (1, 443) = 82.76, *p* < 0.001]. The increase in R^2^ is modest but significant. The modeling procedure was repeated using AV adoption intentions as the dependent variable, yielding a similar pattern of results; results are in Appendix E.

### Summary of Study 1

Our models of AV adoption intention and policy support explain a large amount of variance (78–82%) in these outcomes. Trust in AV technology has a modest yet significant unique influence on AV policy support and adoption intentions, above and beyond a comprehensive set of established predictors. Although competence and integrity were not examined separately in this study, it provides significant evidence of the importance of trust in AV technology in AV adoption decisions. The largest amount of variance in the models is consistently explained by the block with risk perceptions, affect, and perceived benefits of AVs, highlighting the importance of these variables as well.

## Studies 2a and 2b

### Overview

Study 1 supports trust in AV technology as a unique predictor of AV adoption intention and policy support. In Studies 2a and 2b, we begin our exploration of trust as a multi-dimensional construct and examine the extent to which competence and integrity of the manufacturers vs. of the technology itself influence adoption intentions across two risk domains: airplanes and AVs. Traveling by airplane is similar to traveling in an AV in that both modes of transportation are not operated by oneself and thus involve ceding control over one’s safety as a passenger. However, airplanes have a human pilot at the helm – i.e., an agent upon whom integrity is commonly conferred (Siegrist, 2019). Hence, when it comes to traveling by airplane, perceptions of integrity are likely based on both the pilot and the plane itself. Because fully autonomous AVs lack a human operator, people riding in an AV are entrusting the technology itself with their lives and safety, and hence may be more likely to perceive AVs as moral agents with integrity (Waytz et al., 2014; Bigman et al., 2019; Siegrist, 2019). Nor are airplanes emerging technology that remains relatively unfamiliar to the general public. Previous research establishes that people are more familiar with airplanes than AVs (Sintov and Hurst, 2022). Because people are more likely to reason about objects with which they are relatively unfamiliar as moral agents (Epley et al., 2007), people may be more likely to imbue AVs with moral agency than they are airplanes. Thus, we predicted that we would observe an influence of integrity of the technology on adoption intentions in the AV domain but not the air travel domain.

### Methods

#### Procedures

The procedures for Studies 2a and 2b were identical except that Study 2a focused on AVs and Study 2b focused on Airplanes. In each study, respondents completed an online survey on Qualtrics in Spring 2020. The survey took 5-6 min, and participants were compensated $0.80. In both studies, participants read a vignette designed to reduce psychological distance relevant to the domain of focus (see Appendix C), and then completed a series of measures assessing competence and integrity of the respective (i.e., AVs or airplanes) technology and manufacturers, familiarity (knowledge and experience), adoption/use intentions, policy support and demographics. One attention check item (i.e., “Please select ‘disagree’ as your response to this item”) was included.

#### Measures

##### Competence and integrity

For each study, competence and integrity of both the technology (i.e., AV or airplane) as well as the relevant social entity (i.e., AV or airplane manufacturer) were assessed using two-item measures adapted from prior work (Siegrist et al., 2003; Terwel et al., 2009; Liu et al., 2018). Responses were given on a 7-point scale ranging from 1 = strongly disagree to 7 = strongly agree. See Table 2 for the measures used Study 2a. The same questions were adapted for use in Study 2b.

**TABLE 2:** Measures used to assess competence and integrity in autonomous vehicle (AV) technology and manufacturers.

		Trustee	
		Technology	Manufacturer
Trust type	Competence	The AV has the capability to drive well. The AV is a skillful vehicle. (*r* = 0.61)	I would trust the AV manufacturer to do its job well. The AV manufacturer is a competent organization. (*r* = 0.63)
	Integrity	I would trust the AV to do the right thing. I would trust the AV to have good intentions. (*r* = 0.63)	I would trust the AV manufacturer to do the right thing. I would trust the AV manufacturer to have good intentions. (*r* = 0.73)

##### Familiarity

Knowledge was assessed *via* a single item [“In general, how knowledgeable are you about AVs (air travel)?”] measured on a scale from 1 = extremely knowledgeable to 5 = not at all knowledgeable. Similarly, experience was measured with a single item [“How much experience do you have with AVs (air travel)?”], measured on a 5-point scale from 1 = none at all to 5 = a great deal.

##### Other measures

Two items were included to measure adoption intentions {e.g., if the chance arises, how likely are you to ride in an AV [airplane] (measured on 7-point scales)}. The mean was taken to form a scale (AV *r* = 0.75, airplane *r* = 0.58). Demographic variables were assessed using the same measures as Study 1.

#### Pilot study

We conducted a brief pilot study, focused on the AV domain, to validate our measures using a sample of *N* = 866 recruited from Prolific.co. The pilot study suggested acceptable reliability and validity of the measures, as indicated by correlation coefficients for items within each of the scales greater than 0.5. Additionally, correlations between key variables in the study (i.e., Manufacturer Integrity, Manufacturer Competence, Technology Integrity and Technology Competence and Adoption Intentions) were greater than 0.3, indicating construct validity (Hinkle et al., 2003; Carlson and Herdman, 2012).

#### Participants

In the AV domain (Study 2a), we recruited a sample of 869 participants from prolific.co. No changes were made between the pilot study and Study 2a, so the two samples were combined. From our initial sample of 1,735, we dropped 38 participants who failed the attention check and 6 participants who had a missing response for one or more of the key variables. This left us with N_2a_ = 1,691 participants. We conducted a *post hoc* power analysis for a linear multiple regression with 10 predictors. We used the following parameters for the analysis: *f*^2^ = 0.41 (calculated from the regression reported below), α = 0.05, and *N* = 1,691. This analysis revealed that we had 100% power to detect the effect of the four dimensions of trust on the dependent variable. The median age of participants was 29 years old. Just over half of participants identified as women (51%). A majority of participants reported having received a bachelor’s degree or higher (57%). Finally, 63% of the sample identified as at least slightly liberal.

In the airplane domain (Study 2b), we recruited a sample of 871 participants from Prolific.co. From this initial sample, we dropped 14 participants who failed the attention check and 4 participants who had a missing response for one or more of the key variables. This left us with a final sample of N_2b_ = 853 participants. We conducted a *post hoc* power analysis for a linear multiple regression with 10 predictors. We used the following parameters for the analysis: *f*^2^ = 0.10 (calculated from the regression reported below), α = 0.05, and *N* = 853. This analysis revealed that we had 100% power to detect the effect of the four dimensions of trust on the dependent variable. The sample was demographically similar to the AV sample. The median age of participants was 29 years old. Just over half of participants identified as women (52%). A majority of participants reported having received a bachelor’s degree or higher (56%). Finally, 64% of the sample identified as at least slightly liberal.

#### Analyses

In each study, we regressed adoption intentions (or use intentions, in the case of air travel) on the four trust dimensions. We ran the regression models with and without controlling for demographic variables, experience and knowledge (models with covariates shown in Table 3).

**TABLE 3:** Study 2a [autonomous vehicle (AV) domain] regression models predicting adoption intentions.

	Model I	Model II	Model III
Constant	4.92***	6.47***	1.22***
**Demographics**			
Gender (man)	0.80***	0.53***	0.43***
Age	−0.02***	−0.02***	−0.01***
Political Orientation	−0.06**	−0.07**	−0.06**
Education	0.03	–0.002	0.02
**Familiarity**			
Experience		0.15**	0.05
Knowledge		−0.39***	−0.20***
*R^2^ Change*		0.06	
*F* (2, 1682)		59.55	
**Trust**			
Manufacturer Integrity			0.01
Manufacturer Competence			0.17***
Technology Integrity			0.31***
Technology Competence			0.37***
*R^2^ Change*			0.29
*F* (4, 1678)			215.91***
**Omnibus**			
R^2^	0.09	0.15	0.44
F (df)	*F* (4, 1684) = 40.38***	*F* (6, 1682) = 48.65***	*F* (10, 1678) = 130.47***

***p < 0.001, **p < 0.01, *p < 0.05, ^+^*p* < 0.10.

### Study 2a results

Both technology integrity and competence significantly predicted adoption intentions (*p*s < 0.001). In addition, manufacturer competence significantly predicted AV adoption intentions (*p* < 0.001). However, manufacturer integrity did not predict intentions. These results held with and without covariates in the model. In addition, the four dimensions of trust together explained an additional 29% of the variance in adoption intentions, above and beyond the covariates [*F(4, 1,678)* = 215.91, *p* < 0.001]. (See Table 4).

**TABLE 4:** Study 2b (airplane domain) regression models predicting adoption intentions.

	Model I	Model II	Model III
Constant	4.80***	4.90***	2.50***
**Demographics**			
Gender (man)	0.04	0.02	0.01
Age	−0.01*	−0.01***	−0.01*
Political Orientation	0.02	0.02	–0.02
Education	0.21***	0.08*	0.08*
**Familiarity**			
Experience		0.35***	0.34***
Knowledge		−0.17**	−0.15*
*R^2^ Change*		0.13	
*F* (2, 846)		64.58***	
**Trust**			
Manufacturer Integrity			0.05
Manufacturer Competence			0.18**
Technology Integrity			0.05
Technology Competence			0.13**
*R^2^ Change*			0.09
*F* (4, 842)			24.84***
**Omnibus**			
*R* ^2^	0.04	0.17	0.25
*F (df)*	*F* (4, 848) = 9.79***	*F* (6, 846) = 29.03***	*F* (10, 842) = 29.32***

***p < 0.001, **p < 0.01, *p < 0.05, ^+^*p* < 0.10.

### Study 2b results

In Study 2b, the same modeling procedures were applied to the airplane domain. In contrast to Study 2a results, only technology and manufacturer competence significantly predicted use intentions (*p*s < 0.01). Neither technology nor manufacturer integrity significantly predicted use intentions. These results held with and without covariates in the model. In addition, the four dimensions of trust together explained an additional 9% of the variance in adoption intentions, above and beyond the covariates [*F(4, 842)* = 24.84, *p* < 0.001]. (See Table 3).

### Summary of Studies 2a and 2b

Technology integrity significantly predicted adoption intentions for AVs but did not predict use intentions for airplanes. These results held controlling for technology competence, manufacturer competence, and manufacturer integrity, with and without covariates in the model. Findings provide some evidence that people rely on integrity *and* competence in the AV technology when making assessments about AVs. Surprisingly, however, and contrary to what previous TCC work would suggest, the integrity of the manufacturer did not predict intentions in either the AV or airplane domain (Earle et al., 2012; Siegrist, 2019).

## Study 3

### Overview

Studies 2a and 2b provided initial evidence that technology integrity can be a dimension of trust that is uniquely relevant to emerging autonomous technologies such as AVs. In Study 3 (*N* = 938), which was preregistered at [doi: 10.17605/OSF.IO/A7RZT], we evaluated whether messaging strategies can leverage this insight to cultivate public trust in AVs. Specifically, we asked how perceived integrity and competence of (1) an AV and (2) AV manufacturer, communicated through messaging, interact to causally impact public support for AVs. Study 3 uses a 2 (low vs. high integrity) × 2 (low vs. high competence) × 2 (manufacturer vs. technology) between-subjects experimental design. Participants read one of eight (randomly assigned) messages comprised of different elements theorized to increase or decrease perceived competence and/or integrity for (1) an AV manufacturer or (2) AV technology. Then they responded to measures of AV adoption intention, policy support, competence, and integrity of (1) the technology and (2) the manufacturer and demographics (i.e., age, race, ethnicity, gender, political orientation).

Previous theoretical and empirical literature suggests that integrity generally outweighs competence (Earle and Siegrist, 2006; Siegrist et al., 2012). In other words, when it comes to developing trust, it may often be the case that people prefer to know that someone is operating with good intentions than high levels of skill (Liu et al., 2020). Thus, we expected to find a main effect of integrity on AV policy support and adoption intentions, independent of the level of competence. Extending this previous work, which has emphasized the integrity ascribed to the relevant social entity only (e.g., manufacturers in this case), we expected to see this effect for *both* the integrity of the manufacturer and the integrity of the technology. Finally, due to its comparatively weaker role, we expected the effect of competence to depend on the level of integrity, such that competence primarily becomes important when integrity is lacking.

Specifically, we proposed the following pre-registered hypotheses:

H1: We expected to find a significant main effect of integrity-based trust such that participants who read a message high in integrity-based trust would report higher AV policy support and adoption intentions than those who read a message low in integrity-based trust, regardless of the level of competence-based-trust and regardless of whether the message was about the technology or the manufacturer.

H2(a-b): We further expected to find a significant positive main effect of competence-based trust on AV policy support and adoption intentions (H2a). However, we expected this main effect to be qualified by a significant interaction effect such that competence would only positively predict policy support and adoption intentions (H2b) when paired with low integrity-based trust. We expected to find this interaction regardless of whether the message was about the technology or the manufacturer, thus, no three-way interaction was predicted.

### Methods

#### Procedures

Respondents completed an online survey on Qualtrics in Spring 2020 that took approximately 10 min. They were compensated US$1.40. Respondents read one of eight (randomly assigned) messages comprised of different elements theorized to increase or decrease perceived competence and/or integrity for (1) an autonomous vehicle manufacturer or (2) an autonomous vehicle. Then they responded to the survey measures and manipulation check described below. The order of adoption intention and policy support were randomly presented. One attention check item (i.e., “Please select ‘disagree’ as your response to this item”) was included.

#### Measures

AV adoption intention (*r* = 0.74), policy support (α = 0.95) and demographics were assessed using the same measures as were used in the previous studies. Integrity and competence of the AV technology and manufacturer were assessed using the same measures as were used in Study 2a.

#### Manipulation

We created eight messages, one to correspond to each of the eight experimental groups described above. The messages were comprised of different elements theorized to increase or decrease competence-based and integrity-based trust in the AV technology or AV manufacturer. Adapted from recent experimental research (Liu et al., 2020), integrity-based trust was manipulated by describing the AV [manufacturer] as (1) caring (vs. not caring) about passenger safety, (2) open and transparent (vs. not open or transparent), and (3) concerned with public interests (vs. not concerned with public interest). Competence-based trust was manipulated by describing the AV [manufacturer] as (1) skilled/experienced (vs. unskilled/unexperienced), competent/knowledgeable (vs. incompetent/having little knowledge), and (3) technically sound/having relevant technical expertise (vs. unsound/little expertise) (see Appendix D).

#### Participants

We recruited 954 participants from the online platform Prolific.co. This sample was determined through *a priori* power analysis. The power analysis was conducted in G*Power software and set at alpha = 0.05, power = 0.95, effect size *f* = 0.18, df = 10, and number of groups = 8. The relatively small effect size of *f* = 0.18 was estimated from previous literature (Liu et al., 2020). This analysis suggested a sample of 762 participants. We multiplied this number by 1.25 to ensure that we would have sufficient power after accounting for incomplete data. This approach is in line with recent sampling recommendations in the behavioral sciences when the effect size is uncertain (Simonsohn et al., 2014a,b).

The responses of 15 participants were removed for failing the attention check (i.e., “please select “agree” as your response to this item”). This left us with a total sample of 939 participants. The median age of respondents was 30 years old. About half of the sample (50.04%) identified as women. A majority of participants reported having received a bachelor’s degree or higher (56%). Finally, the sample leaned liberal, with 63% of the sample identifying as at least slightly liberal.

#### Manipulation check

Three items were included to measure each of the four dimensions of trust manipulated by the messages: (1) manufacturer competence (Cronbach’s α = 0.92), (2) manufacturer integrity (Cronbach’s α = 0.89), (3) technology competence (Cronbach’s α = 0.92), (4) technology integrity (Cronbach’s α = 0.88).

A series of four 2-way ANOVAs tested whether participants’ ratings on their manipulation check measures corresponded to the messages they were randomly assigned to (i.e., those who read a message high in technology competence should score higher on the technology competence manipulation check measure than those who read a message low in technology competence). The omnibus tests for all ANOVAs were significant (*p* < 0.001). Main effects tests confirmed that participants who read messages high in manufacturer competence (*M* = 5.61, *SE* = 0.08) scored significantly higher on the measure of manufacturer competence than those who read the messages low in manufacturer competence [*M* = 4.21, *SE* = 0.08, *F(1, 469)* = 141.41, *p* < 0.001], those who read messages high in manufacturer integrity (*M* = 5.22, *SE* = 0.09) scored higher on the measure of manufacturer integrity than those who read messages low in manufacturer integrity [*M* = 3.52, *SE* = 0.09, *F*(1, 469 = 185.47, *p* < 0.001], those who read messages high in technology competence (*M* = 5.30, *SE* = 0.09) scored higher on the technology competence measure than those who read messages low in technology competence [*M* = 4.40, *F*(1, 462) = 52.95, *p* < 0.001] and, finally, those who read messages high in technology integrity scored higher on the technology integrity measure (*M* = 5.08, *SE* = 0.10) than those who ready messages low in technology integrity [*M* = 3.93, *SE* = 0.09, *F*(1, 462) = 73.62, *p* < 0.001].

### Results

#### Policy support

**FIGURE 1:**
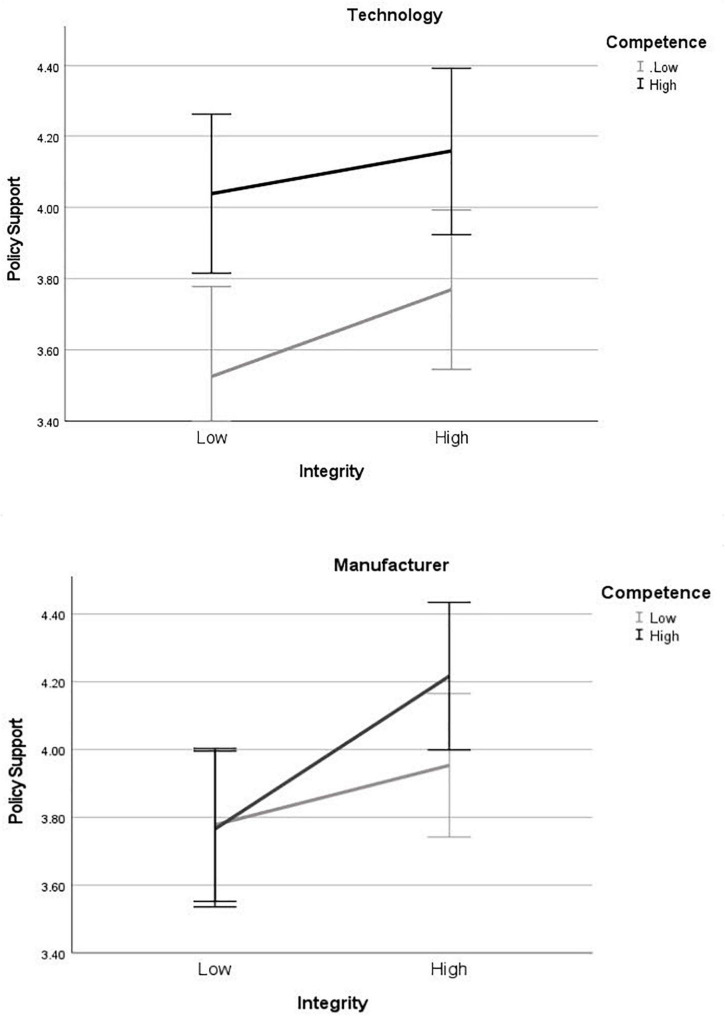
Study 3 policy support ratings for messages about the autonomous vehicle (AV) technology **(top)** and manufacturers **(bottom)**. Error bars show 95% confidence intervals.

**FIGURE 2:**
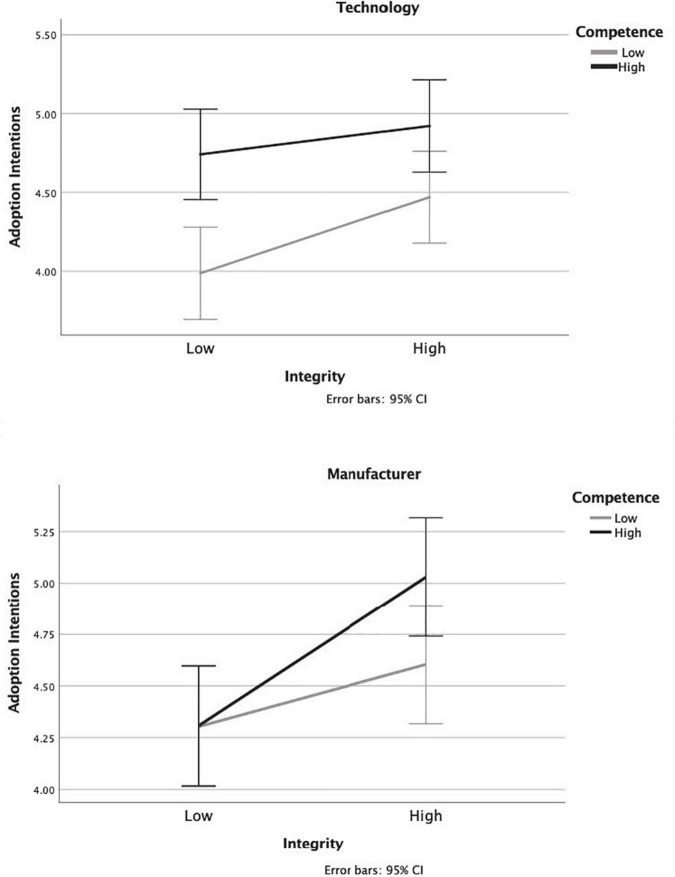
Study 3 adoption intent ratings for messages about the autonomous vehicle (AV) technology **(top)** and manufacturers **(bottom)**. Error bars show 95% confidence intervals.

We ran two three-way ANOVAs (i.e., one for each dependent variable: policy support and adoption intentions) with the three factors being competence (high vs. low), integrity (high vs. low) and entity (manufacturer vs. technology). We followed up with simple effects tests to probe any significant interactions (see Figures 1, 2).

The Omnibus ANOVA with policy support as the DV was significant [*F*(7,931) = 4.13, *p* < 0.001, η^2^ = 0.030]. In support of H1, there was a main effect of integrity, such that those who read high-integrity messages reported stronger policy support (*M* = 4.02, *SE* = 0.06) than those who read messages low-integrity messages [*M* = 3.77, *SE* = 0.06, *F*(1,931) = 9.53, *p* = 0.002, η^2^ = 0.010]. The integrity by entity type interaction was not significant, indicating that, as expected—and in support of the assertion that technology integrity influences adoption decisions— there was a positive effect of integrity regardless of whether the trust manipulated was in the manufacturer or the technology [*F*(1, 931) = 0.717, *p* = 0.397, η^2^ = 0.001]. Further, the integrity by competence interaction was not significant [*F*(1, 931) = 0.255, p = 0.614, η^2^ = 0.000], nor was the three-way interaction [*F*(1,931) = 1.604, *p* = 0.206, η^2^ = 0.002] indicating that the there was an effect of integrity regardless of whether competence was high or low.

In support of H2a, there was a main effect of competence [*F*(1, 931) = 12.37, *p* < 0.001, η^2^ = 0.013]. Contrary to H2b, the insignificant integrity by competence interaction reported above suggests that this effect of competence held regardless of level of integrity. However, we found a significant competence by entity interaction [*F*(1,931) = 4.17, *p* = 0.041, η^2^ = 0.004]. Follow-up simple effects tests revealed an effect of competence only for those who read the technology (vs. manufacturer) message [mean difference = -0.451, *F*(1,931) = 15.34, *p* < 0.001, η^2^ = 0.016]. In contrast, competence did not have an effect on policy support among those who read messages about the manufacturer [mean difference = 0.120, *F*(1,931) = 1.10, *p* = 0.295, η^2^ = 0.001] (see Figure 1).

#### Adoption intentions

The Omnibus ANOVA with adoption intentions as the DV was significant [*F*(7, 931) = 5.53, *p* < 0.001, η^2^ = 0.04]. There was a main effect of integrity such that those who read messages high in integrity-based trust reported higher adoption intentions (*M* = 4.76, *SE* = 0.07) than those who read messages low in integrity-based trust [*M* = 4.34, *SE* = 0.07, *F*(1, 931) = 16.27, *p* < 0.001, η^2^ = 0.017]. The integrity by entity type interaction was not significant indicating that there was a positive effect of integrity regardless of whether the trust manipulated was in the manufacturer or the technology [*F*(1, 931) = 0.74, *p* = 0.39, η^2^ = 0.001]. Further, the integrity by competence interaction was not significant [*F*(1, 931) = 0.08, *p* = 0.777, η^2^ = 0.000], indicating that the there was an effect of integrity regardless of whether competence was high or low. In addition, the three-way interaction did not reach significance [*F* (1,931) = 3.00, *p* = 0.084, η^2^ = 0.003].

The same ANOVAs testing for H1 were used to test H2. There was a main effect of competence-based trust [*F*(1, 931) = 15.33, *p* < 0.001, η^2^ = 0.016]. In this model, the competence by entity type interaction was only marginal [*F*(1,931) = 3.46, *p* = 0.063, η^2^ = 0.004]. However, as with policy support, simple effects tests showed that there was an effect of competence only for those who read the message about the technology [mean difference = 0.60, *F*(1, 931) = 16.55, *p* < 0.001, η^2^ = 0.017]. The level of competence did not have an effect on policy support among those who read messages about the manufacturer [mean difference = 0.22, *F*(1,931) = 2.13, *p* = 0.145, η^2^ = 0.002]. As reported above, the integrity by competence interaction was not significant, nor was the three-way interaction suggesting that this effect of competence held regardless of level of integrity.

### Summary of Study 3

The results of study 3 suggest that overall, messaging that cultivates trust in AVs and AV manufacturers can be an effective strategy for increasing AV policy support and adoption intentions. More specifically, our findings align with previous research in confirming the importance of communicating integrity for increasing public support of an emerging technology (e.g., Liu et al., 2020). However, we add a novel contribution to this literature by finding that in the AV domain, it is important to perceive both the AV manufacturers and the AV technology itself as agents that are operating with integrity. Perceptions of competence can be important too, but only competence of the AV technology, and primarily when integrity associated with the technology is low.^3^

## Discussion

This set of studies expands on previous research that has identified trust as a predictor of consumer acceptance of AVs (Choi and Ji, 2015; Liu et al., 2018). Specifically, we find evidence that trust in AVs explains unique variance in AV policy support and use intentions above and beyond a set of established predictors.

### Contributions to the literature on trust and automation

In addition, extending the TCC model, we find evidence that perceived technology integrity acts as a distinct dimension of trust influencing public support for technology that may be unique to the AV domain or similar autonomous technologies (Earle and Siegrist, 2006; Earle, 2010; Siegrist, 2019). Previous literature in this area has argued that integrity implies agency on the part of the trustee and thus, is only conferred upon a person or a “person-like” entity, such as an institution. Competence, on the other hand, implies objectivity and can be conferred upon an entity such as organization, a person, or an object “who is perceived to have institutional qualities” (Earle et al., 2012, p. 12). Our results from Studies 2a and 2b, however, show that while considerations of technology integrity do not influence decisions about whether to travel by airplane, they do influence decisions about riding in AVs. Prior research on anthropomorphism provides insight into why this might be the case. Specifically, Epley et al. (2007) suggest that people may be more likely to reason about objects as moral agents (i.e., anthropomorphize) when the object is relatively unfamiliar and, thus, is not associated with an existing cognitive framework of understanding (Epley et al., 2007). Applying this idea to the current research, while many people are familiar with airplanes—often through personal experience (e.g., Sintov and Hurst, 2022)—they are less likely to have existing cognitive frameworks for interacting with personal vehicles that operate without a human driver. In addition to familiarity, people are prone to anthropomorphize when the object in question has some type of human-like quality. This factor clearly applies to AVs as—unlike airplanes—the vehicle is moving completely autonomously and interacting with passengers using a human-like voice.

Finally, in terms of competence, the significant entity by competence interaction suggests that it is primarily technology competence that plays a key role in public support for AVs (i.e., manufacturer competence matters less). Another way of putting this is that for social entities, such as manufacturers, high levels of competence cannot make up for low integrity. However, for autonomous technology, competence can make up for low integrity.

### Practical implications for autonomous vehicle expansion

Our findings have practical implications for increasing public acceptance of AVs. When it comes to the AV technology, studies 2a, 2b, and 3 suggest that both competence and integrity considerations factor into decision making. Further, our findings demonstrate that public trust in AVs, cultivated through messaging, can increase AV policy support and adoption intentions.

#### Strengthening perceptions of technology integrity

Perhaps most importantly, we show that perceived integrity of the technology impacts public support for AVs, unlike previous work that has not explored this relationship. Identifying strategies to increase the perceived integrity of the AV technology may be particularly important in light of research demonstrating that people do not want machines—including AVs— to make moral decisions, such as what to do in life-or-death situations (Bigman and Gray, 2018). This aversion to machines making moral decisions holds even when the decisions made by the machine yields positive outcomes, ostensibly because the machine is perceived to lack a full human mind (Bigman and Gray, 2018; Bigman et al., 2019). We argue that enhancing the perceived integrity of the AV may not only advance public support for AVs, but could simultaneously serve to increase people’s comfort with the idea of the technology making morally relevant decisions. Further, it is possible that less aversion to the AV technology making a moral decision may partially explain the relationship between higher perceived integrity of the AV technology and support for AV policy and adoption intentions. Future research should test this proposition.

However, increasing technology integrity is not straightforward. We do not advocate directly telling consumers that the AV is operating with integrity, as this is clearly misleading. Nor do we necessarily suggest that manufacturers go out of their way to anthropomorphize the vehicles, though other research has found that this strategy may be helpful for building trust (Waytz et al., 2014). Rather, our results simply highlight that judgments of the vehicle as a moral agent are likely to occur to some degree and that these judgments will influence decisions regarding AV adoption. Manufacturers can keep this in mind when designing any element of the vehicle that exhibits human-like qualities— such as the tone of voice and language used by the machine when communicating with riders. For example, to show care for its passengers the AV could use the passengers’ names or inquire about their comfort level (e.g., temperature, volume of music). In addition, the AV can demonstrate moral behavior toward others on the road. For example, an AV might be programmed to change lanes in order to give more generous leeway to bicyclists riding along the shoulder, and to communicate to its passengers that it is doing so to show care and consideration for others. Complying with rules and laws is another type of moral behavior (Haidt, 2012). Thus, programming the vehicle to remind passengers of the importance of following the speed limit if they try to speed, for example, can increase perceptions that the vehicle is operating with integrity.

Finally, how the AV communicates with pedestrians and other vehicles on the road can increase perceived integrity of AVs more broadly, even among those who have little or no experience riding in one. Design features to emulate eye contact and other forms of human-like signaling to indicate to others that the AV is aware of them and will stop or slow down to let them pass, as a human driver might do, aligns well with this goal (Olaverri-Monreal, 2020).

#### Strengthening perceptions of technology competence

Technology competence could be strengthened both by improving AV technology and by increasing knowledge of current strengths of AV technology. This can be done, for example, by emphasizing the safety and robustness of AV technology in marketing efforts (Grabner-Kräuter and Kaluscha, 2003). Also, communicating openly and clearly about the actual levels of risk relative to human operated vehicles and about how AV algorithms are continually improving (rather than infallible) can increase public perceptions of technology competence (Shariff et al., 2017).

### Limitations and future directions

This study should be viewed in light of several limitations. First, data were self-reported, and therefore subject to self-report biases. Future work should measure actual AV adoption and policy support. Additionally, our samples were recruited online, and were younger, more educated, and more liberal, compared to the U.S. general population. Hence, results may not extend to other populations or settings, and appropriate caution should be exercised in generalizing the findings. An additional consideration is that we only examined two domains—AVs and airplanes. Future research can test other domains in which a machine may be perceived as a moral agent in order to understand the extent to which imbuing technology with integrity is unique to the AV domain or may extend to other technologies. Next, whereas the sample size for Study 3 was determined using an *a priori* power analysis, Studies 1-2b did not use a power analysis. Future research should base sample size needs on power analyses. Also, in Study 3, although manipulation check results supported the validity of our experimental manipulation, future work would benefit from piloting experimental stimuli with a separate sample. Finally, the scales we used were adapted from previous literature and pre-validated measures, and thus, varied in length across measures (i.e., variously five-, six- and seven-point scales).

## Data availability statement

The original contributions presented in the study are publicly available. This data can be found here: https://doi.org/10.17605/OSF.IO/A7RZT.

## Ethics statement

The studies involving human participants were reviewed and approved by The Ohio State University Institutional Review Board (Protocols 2019E0273, 2020E0497, and 2020E0359). Written informed consent for participation was not required for this study in accordance with the national legislation and the institutional requirements.

## Author contributions

KH: conceptualization, formal analysis, methodology, investigation, writing – original draft, writing – review and editing, and project administration. NS: conceptualization, methodology, investigation, validation, writing – original draft, writing – review and editing, supervision, project administration, and funding acquisition. Both authors contributed to the article and approved the submitted version.

## Figures and Tables

**APPENDIX FIGURE A1:**
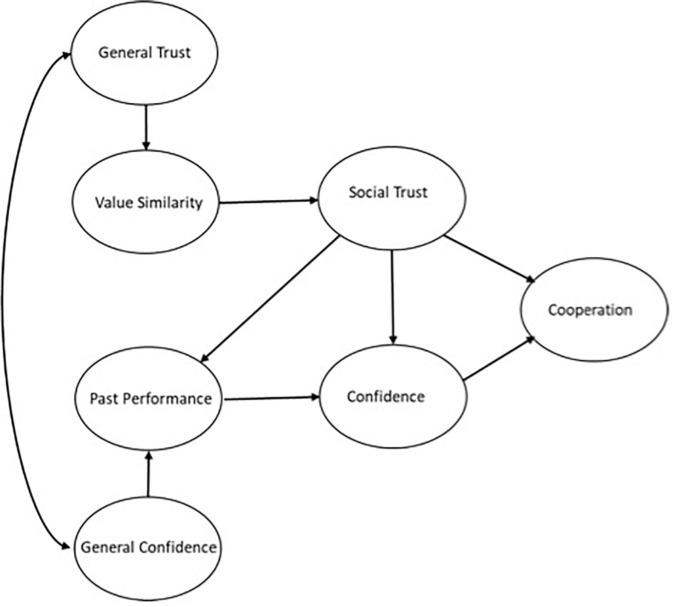
Conceptual model of the trust-confidence-cooperation model. Reproduced from Siegrist et al. (2003), with permission from John Wiley & Sons, Ltd. © 2003 Society for Risk Analysis.

## Footnotes

1 See the Supplemental Information for the results of pre-registered Hypothesis 3, which is not reported here because it is beyond the scope of the current manuscript.

2 Across all study samples fewer than 1.5% of participants choose “other gender”.

3 This pattern of results held when including demographic variables (age, gender, political orientation and education) in the model as covariates.

## Appendix

### Appendix B: AV definition in all studies

“This survey asks several questions about autonomous vehicles (AVs). When we mention AVs, we are referring to self-driving cars that use advanced technologies to sense the surrounding environment and navigate without human input. That is, AVs act on a fully autonomous level, navigating from origin to destination and performing all functions without requiring a human driver”.

### Appendix C: Vignettes used in Studies 2a and 2b

#### Study 2a: AV domain

Imagine that your city has decided to implement a program to test autonomous vehicles on public roads. The test drives are scheduled to begin within the next year and will involve you being assigned your own AV to ride in for the duration of the program.

On the following pages we will ask you a number of questions related to this AV test driving program.

When we mention the AV we are referring to the AV that will be assigned to you for the test drives.

When we mention AV manufacturers we are referring to automobile manufacturers responsible for developing the AVs that will be used for the test drives.

When we mention government authorities we are referring to the authorities that will regulate and supervise the AV test driving program.

#### Study 2b: Airplane domain

Imagine that you are planning a trip that will involve taking a round-trip domestic flight on a commercial airplane. The trip will take place within the next year.

On the following pages we will ask you a number of questions related to this trip.

When we mention the airplane we are referring to the commercial airplane you will be flying on during your trip.

When we mention the airplane manufacturers we are referring to the airplane manufacturers responsible for developing the airplane you will be flying on.

When we mention government authorities we are referring to the authorities that regulate and supervise domestic air travel.

When you respond to these questions please do not consider COVID-19. Rather, when you respond, please consider flying under normal circumstances.

### Appendix D: Study 3 manipulations

#### Manufacturer Messages

##### Introduction (same for all four messages)

Imagine that your city has decided to implement a program to test autonomous vehicles on public roads. The test drives will include opportunities for members of the public to ride as passengers in the vehicles. The following paragraph provides some brief background information on the automobile manufacturer responsible for developing the AVs that will be used for the test drives.

##### Message 1: High integrity, high competence

The automobile manufacturer is known as a company that is honest, open and transparent about its activities. They also have a reputation for having the best interest of the public in mind when developing their vehicles. The automobile manufacturer has been developing AVs for many years. They have extensive knowledge, experience and expertise developing AVs.

##### Message 2: High integrity, low competence

The automobile manufacturer is known as a company that is honest, open and transparent about its activities. They also have a reputation for having the best interest of the public in mind when developing their vehicles. The automobile manufacturer has only recently started to develop AVs. They have limited knowledge, experience, and expertise developing AVs.

You will be able to advance to the next page after 20 s.

##### Message 3: Low integrity, high competence

The automobile manufacturer is not known as a company that is particularly honest, open or transparent about its activities. They also have a reputation for not having the best interest of the public in mind when developing their vehicles. The automobile manufacturer has been developing AVs for many years. They have extensive knowledge, experience and expertise developing AVs.

##### Message 4: Low integrity, low competence

The automobile manufacturer is not known as a company that is particularly honest, open or transparent about its activities. They also have a reputation for not having the best interest of the public in mind when developing their vehicles. The automobile manufacturer has only recently started to develop AVs. They have limited knowledge, experience, and expertise developing AVs.

#### Technology Messages

##### Introduction (same for all four messages)

Imagine that your city has decided to implement a program to test autonomous vehicles on public roads. The test drives will include opportunities for members of the public to ride as passengers in the vehicles. The following paragraph provides some brief background information on the AVs that will be used for the test drives.

##### Message 5: High integrity, high competence

The AVs communicate honestly and openly with their passengers about their activities while on the road. They also have the best interest of the public in mind while driving. AVs have already been driving on public roads in other locations for several years. Overall, AVs are highly skilled, competent and technically sound vehicles.

You will be able to advance to the next page after 20 s.

##### Message 6: High integrity, low competence

The AVs communicate honestly and openly with their passengers about their activities while on the road. They also have the best interest of the public in mind while driving. AVs have only recently begun driving on public roads in other locations. Overall, AVs are not highly skilled, competent or technically sound vehicles.

##### Message 7: Low integrity, high competence

The AVs do not always communicate honestly and openly with their passengers about their activities while on the road. They also don’t always have the best interest of the public in mind while driving. AVs have already been driving on public roads in other locations for several years. Overall, AVs are highly skilled, competent and technically sound vehicles.

You will be able to advance to the next page after 20 s.

##### Message 4: Low integrity, low competence

The AVs do not always communicate honestly and openly with their passengers about their activities while on the road. They also don’t always have the best interest of the public in mind while driving. AVs have only recently begun driving on public roads in other locations. Overall, AVs are not highly skilled, competent or technically sound vehicles.

### Appendix E: Results of Study 1 regression with adoption intentions as the dependent variable

**TABLE 1:** Hierarchical Regression models with autonomous vehicle (AV) adoption intentions as the dependent variable (*N* = 455).

	Model I	Model II	Model III	Model IV
Constant	3.92***	3.37***	0.14	0.09
**Block1:Demographics**				
Gender (man)	0.43***	0.39**	0.02	0.02
Age	−0.02***	−0.02***	–0.001	–0.001
Income	0.28*	0.25*	0.05	0.04
Education	0.15	0.11	0.15*	0.08
Political Orientation^1^	−0.12**	−0.11**	–0.03	–0.02
**Block 2: Familiarity**				
AV experience		0.08	0.06	0.08
AV knowledge		0.75*	0.07	0.003
*R^2^ change*		0.01		
*F* (2,447)		2.46^+^		
**Block 3: Key variables**				
Risk perceptions			−0.09*	0.004
Perceived benefits			0.23***	0.09*
AV-related affect			0.56***	0.33***
*R^2^ change*			0.63	
*F* (3,444)			381.00***	
**Block 4: Trust**				
Trust				0.37***
*R^2^ change*				0.05
*F* (1, 443)				107.79***
**Omnibus**				
R^2^	0.10	0.12	0.75	0.80
F (df)	*F* (5,449) = 11.27***	*F* (7, 447) = 8.81***	*F* (10,444) = 136.18***	*F* (11,443) = 163.38***

****p* < 0.001, ***p* < 0.01, **p* < 0.05, ^+^*p* < 0.10. ^1^ Political orientation was measured on a 7-point scale with 1 = Extremely liberal and 7 = extremely conservative.
