# Supplementary material for: Trusting autonomous vehicles as moral agents improves related policy support
Source: Front Psychol. 2022 Oct 20;13:976023. doi: 10.3389/fpsyg.2022.976023 (PMC9632851; doi:10.3389/fpsyg.2022.976023)
Supplement: Supplementary file 1 [file Data_Sheet_1.docx]

**Supplemental Information**

Below, we report the results of the following pre-registered hypotheses ([10.17605/OSF.IO/A7RZT](https://doi.org/10.17605/OSF.IO/A7RZT)), which was beyond the scope of the main manuscript:

H3(a-j): We expect perceptions of AV-related risks and benefits to mediate the relationship between trust and both AV policy support and AV adoption intentions. Specifically, we expect that higher trust will lead to lower perceptions of risk (H3a), which in turn will be associated with higher AV policy support (H3b) and intentions (H3c). In addition, higher trust will lead to higher perceived benefits (H3d), which in in turn will be associated with higher AV policy support (H3e) and adoption intentions (H3f). Further, we expect to observe significant indirect effects of trust on AV policy support and adoption intentions through perceived risk and benefits (indirect effects, Hg-Hj).

All hypotheses were supported. The results of mediation models with perceived benefits and risk mediating the relationship between the integrity of the technology and public acceptance of AVs are reported below.

**Figure S1.** *Unstandardized path coefficients for mediation model with intentions as the dependent variable*


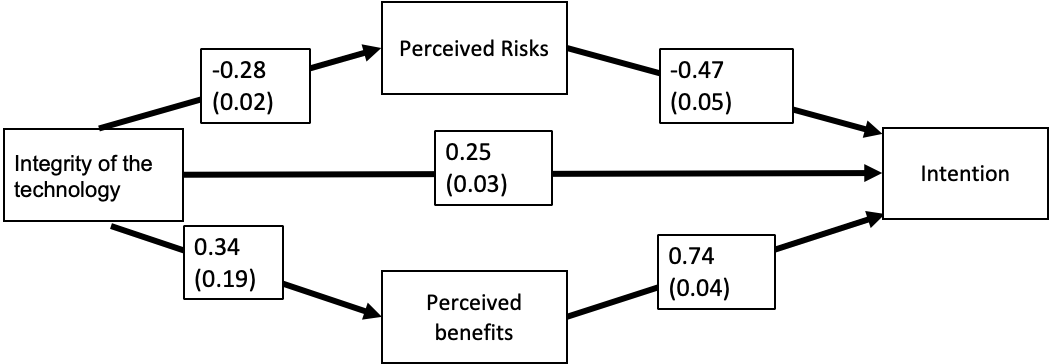


Figure Note. Bold paths indicate significance of the regression coefficient based on 95% bootstrap CIs.

**Figure S2.** *Unstandardized path coefficients for mediation model with policy support as the dependent variable*

**
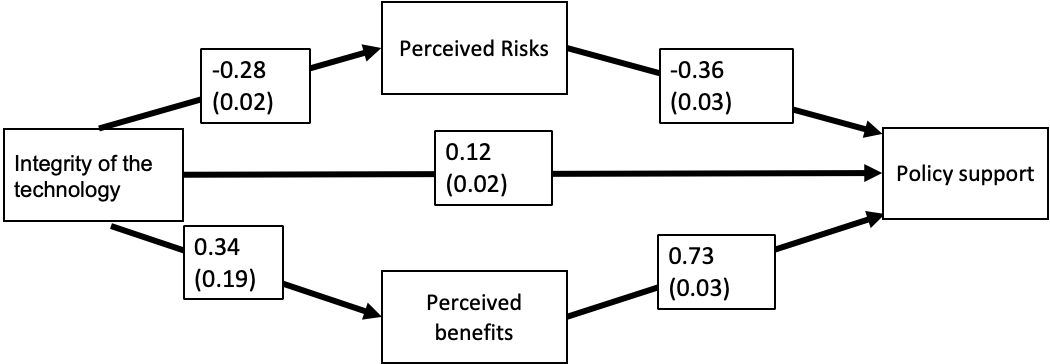
**

**Table S1.** *Summary of mediation model path coefficients, indirect effects, direct effects, and total effects*

| Independent variable (IV) | Mediating variable (M) | Dependent variable (DV) | Effect of IV on M (*a*) | Effect of M on DV (*b*) | Direct effect (*c’*) | Indirect effect  (*ab*) 95% CI | | Total effect (*c*) |
| --- | --- | --- | --- | --- | --- | --- | --- | --- |
| Tech Integrity | Risks | Intentions | **-0.28** | **-0.47** | **0.25** | **0.13** | (0.10, 0.16) | **0.63** |
| Tech Integrity | Benefits | Intentions | **0.34** | **0.74** | **0.25** | **0.25** | (0.22, 0.29) | **0.63** |
| Tech Integrity | Risks | Policy support | **-0.28** | **-0.36** | **0.12** | **0.10** | (0.08, 0.12) | **0.46** |
| Integrity | Benefits | Policy support | **0.34** | **0.73** | **0.12** | **0.25** | (0.22, 0.28) | **0.46** |

Table Note. CI=confidence interval. 5,000 bootstraps. Path coefficients are unstandardized effects. Bolded numbers indicate significance of 95% percentile bootstrap CI.
